# Sex Hormones in Autism: Androgens and Estrogens Differentially and Reciprocally Regulate RORA, a Novel Candidate Gene for Autism

**DOI:** 10.1371/journal.pone.0017116

**Published:** 2011-02-16

**Authors:** Tewarit Sarachana, Minyi Xu, Ray-Chang Wu, Valerie W. Hu

**Affiliations:** Department of Biochemistry and Molecular Biology, The George Washington University Medical Center, Washington, D.C., United States of America; National Institute on Aging Intramural Research Program, United States of America

## Abstract

Autism, a pervasive neurodevelopmental disorder manifested by deficits in social behavior and interpersonal communication, and by stereotyped, repetitive behaviors, is inexplicably biased towards males by a ratio of ∼4∶1, with no clear understanding of whether or how the sex hormones may play a role in autism susceptibility. Here, we show that male and female hormones differentially regulate the expression of a novel autism candidate gene, retinoic acid-related orphan receptor-alpha (RORA) in a neuronal cell line, SH-SY5Y. In addition, we demonstrate that RORA transcriptionally regulates aromatase, an enzyme that converts testosterone to estrogen. We further show that aromatase protein is significantly reduced in the frontal cortex of autistic subjects relative to sex- and age-matched controls, and is strongly correlated with RORA protein levels in the brain. These results indicate that RORA has the potential to be under both negative and positive feedback regulation by male and female hormones, respectively, through one of its transcriptional targets, aromatase, and further suggest a mechanism for introducing sex bias in autism.

## Introduction

Autism refers to a spectrum of neurodevelopmental disorders with a prevalence of ∼1∶110 that are characterized by deficits in social understanding and interactions, aberrant language development and/or usage, and repetitive, stereotyped behaviors, often with restricted interests[1], [2]. The disorder is inexplicably biased towards males by a ratio of at least 4∶1, prompting theories that fetal or perinatal exposure to elevated levels of male hormones may increase susceptibility towards autism[3]. Despite some evidence linking elevated fetal testosterone levels in amniotic fluid to autistic symptomatology[4], [5] and increasing rightward asymmetry of the corpus callosum[6], (which is known to be aberrant in autism[7]) and our own studies which have identified deregulated genes involved in androgen biosynthesis as well as higher testosterone levels in lymphoblastoid cells from autistic individuals [8], no sex hormone-sensitive candidate genes for autism have been reported. At present, there is still no clear understanding of the molecular mechanisms through which the sex hormones may play a role in autism susceptibility.

We have recently identified a novel autism candidate gene, retinoic acid-related (RAR) orphan receptor-alpha (RORA)[9], which is a hormone-dependent transcription factor. In brief, our combined studies have demonstrated: a) reduced expression of RORA in lymphoblastoid cell lines (LCL) derived from autistic individuals[10], [11]; b) increased methylation and reduced protein expression of RORA in the LCL[9]; and c) decreased expression of RORA protein in autistic brain[9]. Together, these results link molecular changes in RORA in peripheral cells to molecular pathology in the brain of autistic individuals. These findings are particularly relevant to ASD as RORA is involved in several key processes negatively impacted in autism, including Purkinje cell differentiation[12], cerebellar development[13], [14], protection of neurons against oxidative stress[15], suppression of inflammation[16], and regulation of circadian rhythm[17]. Behavioral studies on the RORA-deficient *staggerer* (RORA^+/sg^) mouse, primarily used as a model to study ataxia and dystonia[13], further show that RORA is also associated with restricted behaviors reminiscent of ASD, such as perseverative tendencies[18], limited maze patrolling[19], anomalous object exploration[20] as well as deficits in spatial learning[21]. Although there are currently no reported studies on the social behaviors of *staggerer* mice, it is clear that RORA is associated with at least some of the symptomatology and pathology of ASD.

Here, we show that the level of RORA expression can be regulated by both male and female hormones through their respective receptors, and that one of its transcriptional targets is CYP19A1 (aromatase), an enzyme responsible for the conversion of testosterone to estrogen. We further show that the amount of aromatase protein in brain tissues from autistic and nonautistic donors correlates with the amount of RORA protein, with a statistically significant reduction of both RORA and aromatase in autistic tissues. We thus propose that in ASD, down-regulation of RORA is exacerbated by a negative feedback mechanism involving reduction of aromatase that can result in build-up of its substrate testosterone which, in turn, can further suppress RORA expression.

## Results

### Male and female hormones oppositely regulate RORA expression in human neuroblastoma cells

**Figure 1:**
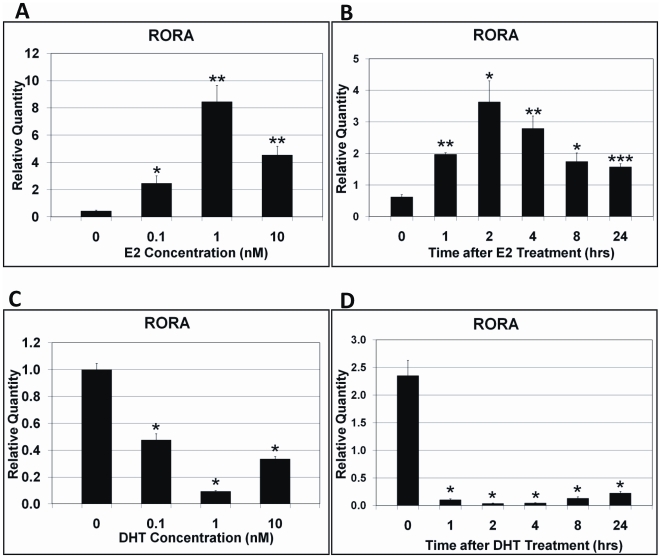
Effect of sex hormones on RORA expression in SH-SY5Y cells. (A) Dose-response to estradiol. The cells were treated with 0.1, 1, or 10 nM 17β-estradiol (E2) for 2 hrs and RORA expression was measured by qRT-PCR analyses (n = 3 per treatment group). (B) Time-course of response to estradiol. The cells were treated with 1 nM 17β-estradiol and qRT-PCR analyses (n = 3 per time point) were conducted to determine RORA expression at different times after hormone addition. (C) Dose-response to DHT. The cells were treated with 0.1, 1, or 10 nM DHT for 2 hrs and RORA expression was measured by qRT-PCR analyses (n = 3 per treatment group). (D) Time-course of response to DHT. The cells were treated with 10 nM DHT and qRT-PCR analyses (n = 3 per time point) were conducted to determine RORA expression at different times after hormone addition. Error bars indicate SEM (*P<0.05, **P<0.01, ***P<0.005, versus mock-treatment control).

Because RORA is a hormone-dependent transcription factor that is involved in a variety of functions impacted in autism, we sought to investigate the effects of both male and female hormones on RORA expression. Inasmuch as estrogens are known to have neuroprotective effects on the brain and RORA has been reported to be neuroprotective against oxidative stress [15] and inflammation [16], we first treated the human neuroblastoma cell line SH-SY5Y with different concentrations of 17β-estradiol, and measured RORA expression by qRT-PCR analyses. The estradiol treatment significantly enhanced RORA expression, and the greatest upregulation (∼15-fold) of RORA was observed when the cells were treated with 1 nM estradiol (Fig. 1A). We also conducted a time-course experiment to investigate changes in RORA expression at different time points after estradiol exposure. The time-course study revealed that RORA expression was highest at 2 hrs after estradiol treatment, and the upregulation of RORA could be observed as long as 24 hrs after the treatment (Fig. 1B).

To determine whether male hormones (androgens) also influence RORA expression, dose-effect and time-course experiments using dihydrotestosterone (DHT) were performed in the same manner as the aforementioned estradiol treatment. Interestingly, in contrast to estrogen treatment, DHT treatment caused a decrease in RORA expression compared with mock treatment (Fig. 1C). A time-course study showed that the greatest suppression of RORA was observed between 2 and 4 hrs, and downregulation of RORA could be observed as long as 24 hrs after DHT treatment (Fig. 1D). This unexpected result (that is, decrease in RORA expression) led us to investigate whether or not RORA might be a transcriptional target of both estrogen receptor (ER) and androgen receptor (AR) by chromatin immunoprecipitation (ChIP)-PCR analyses.

### RORA is a potential transcriptional target of androgen and estrogen receptors

**Figure 2:**
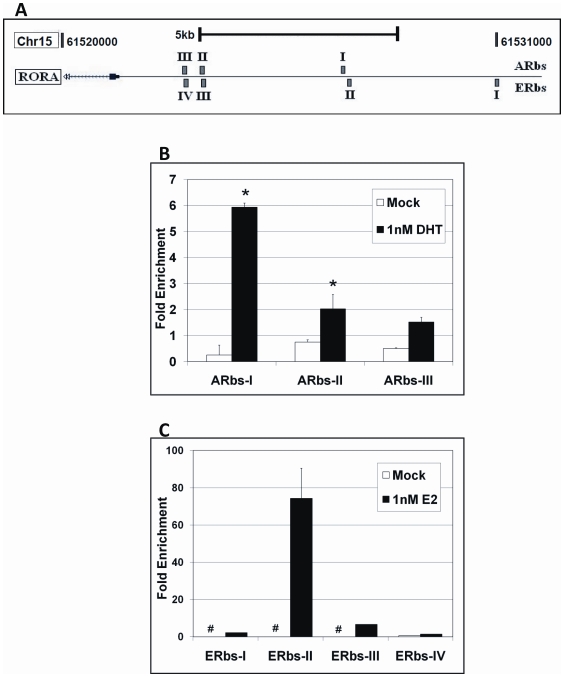
RORA is a potential transcriptional target of both AR and ER. (A) Schematic diagram showing the upstream region of the RORA gene (edited from the UCSC Genome Browser). Potential AR and ER binding sites are labeled (ARbs  =  AR potential binding site, ERbs  =  ER potential binding site). (B) Chromatin Immunoprecipitation followed by qPCR (ChIP-qPCR) analyses of AR potential binding sites on the RORA gene promoter region. Sonicated chromatin from SH-SY5Y cells treated with 1 nM DHT for 2 hrs was immunoprecipitated (IP) using anti-AR or IgG antibody. Parallel samples were mock-treated with the hormone delivery vehicle, ethanol. The enrichment of each AR binding site in anti-AR-IP DNA was determined by qPCR analyses (n = 3), and normalized to the enrichment in IgG-IP DNA (control). Error bars indicate SEM (*P<0.01 versus mock-treatment control). (C) ChIP-qPCR analyses of ERα potential binding sites on the RORA gene promoter region. Sonicated chromatin from SH-SY5Y cells treated with 1 nM 17β-estradiol (or vehicle) for 2 hrs was immunoprecipitated using anti-ERα or control IgG antibody. The enrichment of each ERα binding site in anti-ERα-IP DNA was determined by qPCR analyses (n = 3), and normalized to the enrichment in IgG-IP DNA. Error bars indicate SEM (**^#^**Undetectable amount of ERα binding sites in the mock-treatment control).

DHT and estradiol are known to exert their transcriptional regulatory functions through androgen receptor (AR) and estrogen receptor alpha (ERα), respectively. To investigate whether RORA might be a direct transcriptional target of either AR or ERα, we first identified potential binding sites for AR and ERα within 10 kb upstream of the RORA transcription start site using JASPAR, PROMO 3.0, and SABioscience ChampionChIP Real-Time PCR databases[22]–[24]. Three potential AR binding sites (namely ARbs I-III) and four potential ERα binding sites (namely ERbs I-IV) were selected for ChIP-qPCR analyses (Fig. 2A). The list of all potential binding sites and their genomic locations is shown in Table S1.

Neuroblastoma cells, treated with 1 nM DHT or 1 nM estradiol, were employed for the ChIP assays using anti-AR or anti-ERα antibody, respectively. Interestingly, the enrichment of the AR binding sites in anti-AR-IP DNA was significantly increased when the cells were treated with DHT, compared with mock treatment (Fig. 2B). The highest enrichment was observed in the ARbs-I region. Similarly, we found a significant increase in enrichment of the ERα binding sites in anti-ERα-IP DNA when the cells were treated with estradiol (Fig. 2C). The ERbs-II site exhibited the greatest enrichment (∼70-fold), compared with the other potential ER binding sites. These data indicate that RORA is likely a direct transcriptional target of both AR and ERα, with DHT and estradiol treatment significantly increasing the binding of AR and ER, respectively, to the RORA promoter region.

### Aromatase is a transcriptional target of RORA

**Figure 3:**
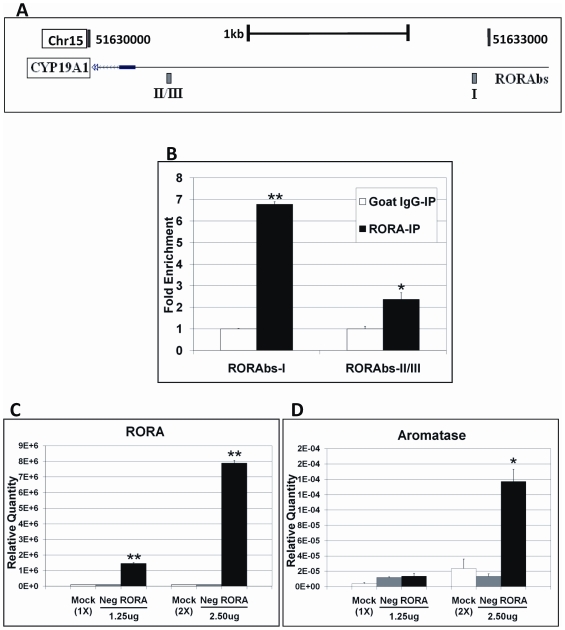
Aromatase is a potential transcriptional target of RORA. (A) Schematic diagram showing the upstream region of the aromatase gene (edited from the UCSC Genome Browser). Potential RORA binding sites are labeled (RORAbs  =  RORA potential binding sites). Because sites II and III are so close to each other, a single pair of primers was designed to cover both sites. (B) ChIP-qPCR analyses of potential binding sites for RORA on the aromatase gene promoter region. Sonicated chromatin from SH-SY5Y cells was immunoprecipitated using anti-RORA or control IgG antibody. The enrichment of each RORA binding site in anti-RORA-IP DNA was determined by qPCR analyses (n = 3). Error bars indicate SEM (*P<0.05, **P<0.001, versus the enrichment in IgG-IP DNA). **Aromatase expression is enhanced by RORA overexpression**. SH-SY5Y cells were transfected with 1.25 µg or 2.50 µg pSG5.HA-RORA plasmid or empty plasmid for 24 hrs, and qRT-PCR analyses (n = 3) were conducted to measure RORA and aromatase expression (Mock  =  mock-treatment control, Neg  =  empty plasmid, RORA  =  pSG5.HA-RORA plasmid). (C) Relative quantity of RORA transcript levels in RORA-transfected cells which is increased by more than a factor of 10^5^ relative to the controls. (**P<0.001 versus the empty plasmid control) (D) Relative quantity of aromatase transcript levels in RORA-transfected cells. Error bars indicate SEM (*P<0.05 versus the empty plasmid control).

Aromatase (CYP19A1), a member of the cytochrome P450 family of proteins, is responsible for estradiol biosynthesis. This protein is the key hydroxylating enzyme which converts androstenedione to estrone and testosterone to estradiol. Aromatase is therefore considered a crucial protein in regulating levels of male and female sex hormones in various tissues, including gonads, adipose tissue, placenta, and brain. Using the JASPAR, PROMO3.0, and ChampionChIP databases, we found 3 potential RORA binding sites (RORAbs I-III) within 2 kb upstream of the aromatase gene (Fig. 3A, Table S1). Two sites, one of which is a combination of adjacent sites II and III, were selected for ChIP-qPCR analyses. To determine whether RORA could directly bind to these potential binding sites on the aromatase gene, ChIP-qPCR analyses were conducted using anti-RORA antibody. A significant enrichment of the RORAbs-I region was observed in the anti-RORA-IP DNA (Fig. 3B), indicating that aromatase is likely to be a direct transcriptional target of RORA.

Functional analysis of RORA was conducted to further demonstrate that aromatase is transcriptionally regulated by RORA. Human neuroblastoma cells were transfected with a pSG5.HA vector containing the DNA-binding and transactivation sequence of RORA (pSG5.HA-RORA), and qRT-PCR analyses were performed to determine the amount of RORA and aromatase transcripts in the transfected cells 24 hrs after transfection. A significant increase in RORA transcript level was observed in the cells transfected with the plasmid, indicating that the transfection was successful (Fig. 3C). Interestingly, overexpression of RORA was accompanied by a significant increase (∼10-fold) in aromatase transcript level (Fig. 3D). This finding thus demonstrates that aromatase expression is regulated by RORA.

### Aromatase protein is decreased and correlated with RORA protein level in postmortem brain tissues of autistic individuals

**Figure 4:**
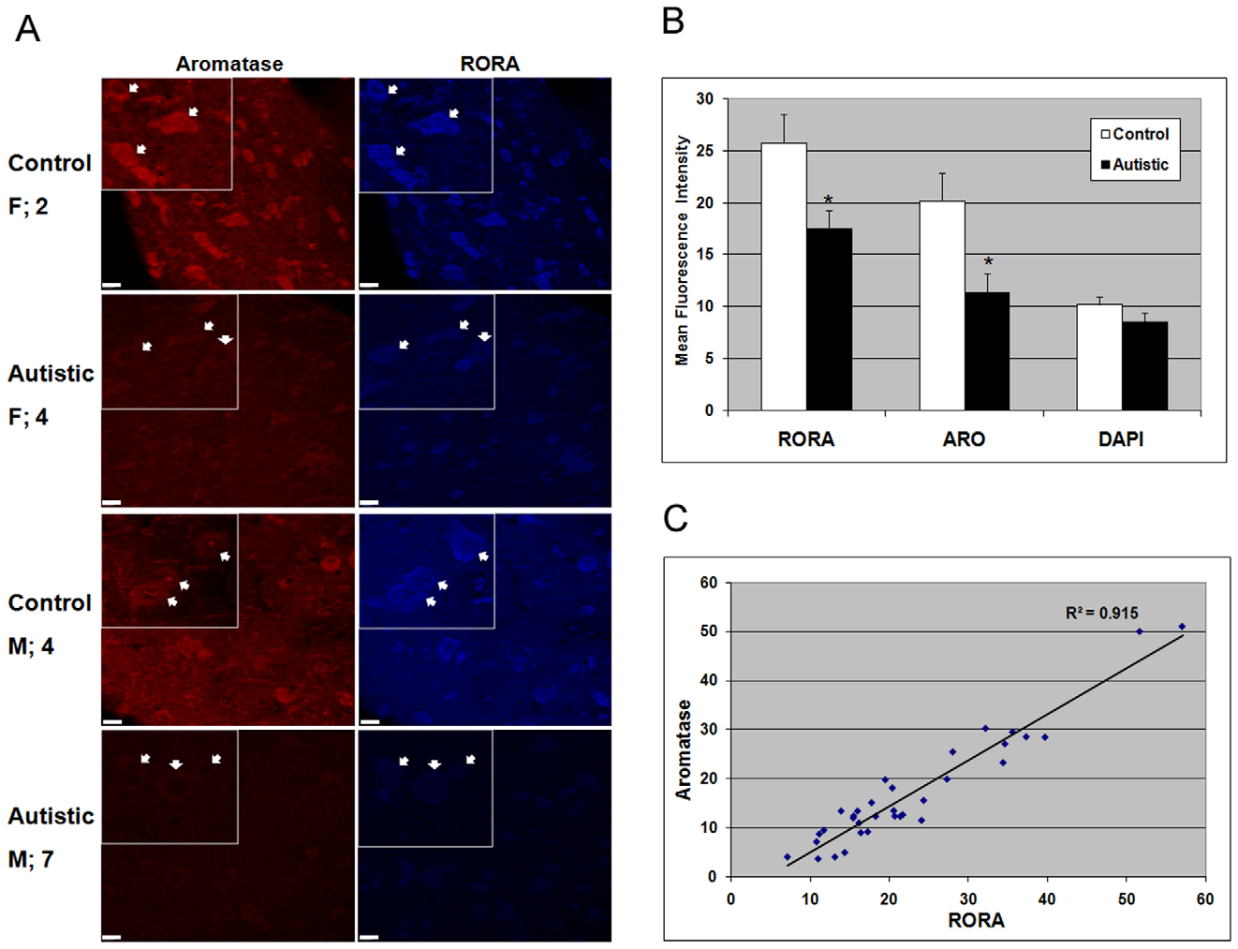
RORA and aromatase proteins are decreased in postmortem brain tissues from autistic subjects. A tissue array containing postmortem frontal cortex specimen from 12 autistic individuals and 22 age- and sex-matched controls was co-immunolabeled for aromatase (red), RORA (blue), and MAP2, and counterstained with DAPI to reveal nuclei (see Fig. S1 for example of quadruple staining). (A) Representative images from confocal immunofluorescence analyses of the frontal cortex tissue arrays. Arrows mark neurons in the frontal cortex. Scale bars, 20 µm. F: female; M: male; #'s indicate the respective ages of the subjects from whom the brain tissues were obtained. (B) Quantitative analyses of confocal fluorescence images of RORA, aromatase, and DAPI. Frontal cortex neurons expressing MAP2 protein were selected according to its green immunofluorescence (shown in Fig. S1), and fluorescence signal intensity for RORA, aromatase (ARO), and DAPI, in the neurons were extracted using image analysis software and normalized by background subtraction. For each sample, ∼40–50 neurons were selected for quantification of RORA and aromatase fluorescence in a “sample blind” fashion such that the identities of the samples were unknown to the person performing the analyses. Error bars indicate SEM (*P<0.05 versus the control samples). (C) Correlation analysis of RORA and aromatase protein expression in the frontal cortex neurons.

Our laboratory has recently demonstrated the reduction of RORA protein in the frontal cortex and the cerebellum of autistic subjects relative to that of control subjects by immunohistochemical analyses of tissue arrays[9]. In this study, confocal immunofluorescence analyses of tissue arrays containing postmortem frontal cortex from autistic individuals and sex- and age-matched controls were conducted to quantify the expression levels of RORA and aromatase protein in the brain. The tissue arrays were co-immunolabeled for RORA, aromatase, and the neuronal marker MAP2, and then were counter-stained with DAPI to visualize the nucleus of the cells. Notably, both RORA and aromatase proteins were significantly reduced in frontal cortex neurons from autistic subjects compared with those from sex- and age-matched controls (Fig. 4A, B). Moreover, expression of aromatase protein in the neurons was also strongly correlated with RORA expression (R^2^ = 0.915; Fig. 4C).

## Discussion

The results of this study suggest that the expression of RORA is inversely modulated by male and female sex hormones, with DHT and estradiol increasing the binding of AR and ER, respectively, to the RORA promoter region. Interestingly, estradiol enhances RORA expression, whereas DHT represses expression of RORA. The specific mechanism and circumstances through which AR and ER regulate RORA in opposite directions are unknown and require further study. Although these hormonal effects were observed in the SH-SY5Y neuroblastoma cell line which is a widely used neuronal cell model, this line may not replicate all responses of primary neurons. Thus, it would also be important to study the effects of sex hormones on RORA expression and interactions in primary neurons, using an appropriate animal model. In this respect, the *accelerated Purkinje cell loss* observed during aging in male *staggerer* (RORA^+/sg^) mice vs. female *staggerer* mice supports the concept of interaction between the sex hormones and RORA, with the male mice being more susceptible to RORA deficiency [25]. By comparison, no gender-related differences in the progression of Purkinje cell loss were observed in wild-type (RORA^+/+^) mice.

**Figure 5:**
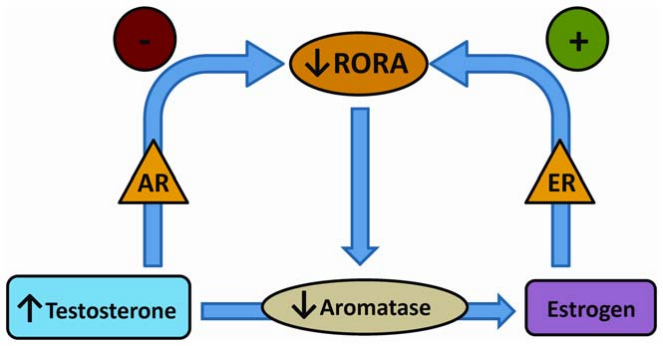
A model for reciprocal hormonal effects on RORA. The schematic illustrates a mechanism through which the observed reduction in RORA in autistic brain may lead to increased testosterone levels through downregulation of aromatase. Through AR, testosterone negatively modulates RORA, whereas estrogen upregulates RORA through ER.

We also show that one of the transcriptional targets of RORA is aromatase, which is a crucial enzyme in the biosynthesis of estrogen from testosterone. It is noteworthy that both RORA and aromatase proteins are decreased in the frontal cortex of autistic subjects, and that the level of aromatase protein is strongly correlated with the level of RORA protein in the brain tissues. We therefore propose that the reduction of RORA observed in autism is exacerbated by a negative feedback mechanism involving decreased aromatase level, which further causes accumulation of its substrate, testosterone, and reduction of its product, estradiol. Testosterone and estradiol respectively exhibit negative and positive feedback regulation of RORA expression as illustrated in **Fig. 5**, which summarizes the principal findings of this study. Thus, a deficiency in RORA in autistic brain is expected to be further aggravated by increased levels of testosterone due to suppression of aromatase, a transcriptional target of RORA.

These results provide a molecular mechanism whereby testosterone may be increased in some cases of autism and further support for the relevance of RORA as a candidate gene for autism. Indeed, RORA's likely involvement in the balance between male and female hormones in brain tissues through regulation of aromatase transcription, coupled with its critical roles in Purkinje cell differentiation and cerebellar development as well as in neuroprotection against inflammation and oxidative stress, explains at least some of the pathology observed in autism. However, we note that not all samples from autistic individuals are deficient in RORA, as demonstrated by our previous studies with lymphoblastoid cell lines and brain tissues[9], [10]. On the other hand, we observed reductions in RORA in brain tissues from both male and female subjects with ASD, suggesting that RORA deficiency is not gender-specific. Interestingly, RORA and ER share a consensus binding site on DNA (AGGTCA) and consequently common target genes. The existence of shared gene targets may explain why females, with higher levels of estrogens, are less susceptible to autism. That is, estrogens may not only protect females against autism by increasing the level of RORA expression, but also by inducing shared target genes of RORA through ER, thus compensating in part for RORA deficiency.

In summary, we provide the first description of a candidate gene for autism that is responsive to both male and female sex hormones. We also demonstrate that a deficiency in this gene, RORA, can potentially result not only in the increase in testosterone levels, but also in further suppression of itself, through negative feedback regulation which is mediated through its transcriptional target, aromatase, which converts male hormones to estrogens. Finally, we demonstrate the correlated decrease of both RORA and aromatase protein levels in autistic brain, further strengthening support for RORA as a critical candidate gene for autism susceptibility.

## Materials and Methods

### Cell culture

The human neuroblastoma cells SH-SY5Y (ATCC, Manassas, VA) were cultured in 1∶1 Modified Eagle's Medium (MEM) and Ham's F12 media (MediaTech, Manassas, VA) supplemented with 15% (v/v) fetal bovine serum (Atlanta Biological) and 1% penicillin/streptomycin (P/S; MediaTech). Cells were maintained at 37°C with 5% CO_2_, and split 1∶2 every 3–4 days when the cells reached ∼80% confluency. For harvesting, the cells were treated with trypsin-EDTA (MediaTech) for 2–3 min to release them from the surface of the culture flask, followed by the addition of media to inactivate the trypsin. The detached cells were then pelleted by spinning at 2,000 rpm for 5 min at 4°C and gently washed twice with cold PBS.

### Hormone treatment

For hormone treatment, SH-SY5Y cells were carefully washed twice *in situ* with phenol red-free 1∶1 DMEM/F12 media (MediaTech) supplemented with 15% charcoal dextran-treated serum (Atlanta Biologicals, Lawrenceville, GA) and 1% P/S, and then cultured in the phenol red-free medium for 24 hours to deplete the medium of exogenous hormones. Lyophilized 4,5α-dihydrotestosterone (DHT) and 17β-estradiol (Sigma-Aldrich, St. Louis, MO) were diluted with absolute ethanol to make 10 µM DHT and 1 µM estradiol stock solutions which were then used after appropriate dilutions for all hormone treatment experiments. In the dose-response studies, the cells were treated with 0.1 nM, 1 nM, and 10 nM (final concentration) of DHT or estradiol for 2 hrs. In the time course study, cells were treated with 10 nM DHT or 1 nM estradiol, and harvested at 1 hr, 2 hrs, 4 hrs, 8 hrs, and 24 hrs after treatment. Cells treated with an equal volume of vehicle (absolute ethanol) were used as a mock-treatment control for both experiments.

### Plasmid transfection

The DNA sequence coding for the DNA binding and transcription activation sites of human RORA was cloned into the pSG5.HA expression vector and confirmed by sequencing. To enhance RORA expression in the SH-SY5Y neuroblastoma cells, the plasmid was transfected into the cells using Lipofectamine LTX and PLUS reagents (Invitrogen, Carlsbad, CA) according to the manufacturer's protocol. Briefly, SH-SY5Y cells were plated in a 6-well culture plate containing complete growth media without antibiotics (approximately 2.5×10^5^ cells per well) and incubated for 24–36 hrs at 37°C and 5% CO2 until the cell density was 50–60% confluent. The plasmid (1.25–2.50 µg) was diluted in 500 µl of Opti-MEM I Reduced Serum Medium (Invitrogen) and 1.25 µl of PLUS reagent was added to the diluted plasmid solution. Lipofectamine LTX (25 µl) was then added to the plasmid solution and incubated for 25 min at room temperature to form plasmid-Lipofectamine LTX-PLUS complexes. The growth medium was aspirated without disturbing the cells at the bottom of the plate, and 500 µl of the plasmid-Lipofectamine LTX-PLUS complexes were added directly to each well. The cells were then incubated with the complexes at 37°C and 5% CO2 for 24 hrs before harvest. For the negative control, the empty plasmid was transfected into the cells in the same manner as the RORA plasmid. Mock treatment without plasmid was also conducted to serve as an additional negative control.

### Quantitative RT-PCR analyses

Quantitative RT-PCR analyses were performed as described [8]. Briefly, total RNA was isolated from the cells using the TRIzol (Invitrogen) isolation method, and cDNA was synthesized using iScript cDNA Synthesis Kit (BioRad, Hercules, CA) according to the manufacturer's protocols. The reaction was incubated at 25°C for 5 min, followed by 42°C for 30 min, and ending with 85°C for 5 min. After reverse transcription, the cDNA reaction was diluted to a volume of 50 µl with nuclease-free water and used as a template for qPCR analyses. Real-time PCR analyses were conducted using ABI Prism 7300 Sequence Detection System (Applied Biosystems, Foster City, CA). Primers for qRT-PCR analyses designed by Primer 3 are listed in Table S2.

### Identification of potential binding sites for AR, ER, and RORA

Putative binding sites for the transcription factors were identified using PROMO 3.0, a web-based program that employs the TRANSFAC database version 8.3 to construct specific binding site weight matrices for prediction of transcription factor binding sites [23], [24]. The putative binding sites were then further validated using JASPAR and ChampionChIP databases [22], and carefully selected for ChIP-qPCR analyses. For AR and ER binding sites on RORA gene, the DNA sequence of RORA plus 10 kb upstream was obtained from the UCSC Genome Browser Database, and analyzed by PROMO3.0. A total of 3 AR binding sites and 4 ER binding sites were selected for ChIP-qPCR analyses. For RORA binding sites on the aromatase gene, the DNA sequence of aromatase (CYP19A1) plus 10 kb upstream was obtained from the UCSC Genome Browser and analyzed in the same manner as the RORA DNA sequence. A total of 3 RORA binding sites on aromatase were selected for ChIP-qPCR analyses. All of the potential binding sites are listed in Table S1.

### Chromatin immunoprecipitation-qPCR (ChIP-qPCR) analyses

Chromatin immunoprecipitation (ChIP) was performed using the Millipore EZ-ChIP Chromatin Immunoprecipitation Kit (Millipore, Billerica, MA) according to the manufacturer's protocol. Briefly, the SH-SY5Y neuroblastoma cells were cultured as previously described and treated with DHT or estradiol for 2 hrs. The cells were then treated with 37% formaldehyde for exactly 10 min to crosslink the proteins to the DNA. The crosslinked cells were transferred to a pre-chilled centrifuge tube, and nuclear extraction was performed using the Active Motif Nuclear Extract Kit (Active Motif, Carlsbad, CA) according to the manufacturer's protocol. Nuclear pellets were resuspended in the SDS Lysis Buffer, and were sonicated on wet ice to shear the chromatin to 200–1,000 bp. The sonication was conducted in a cold room with 25 sets of 15-second pulses using a Heat Systems-Ultrasonics W-380 sonicator (Heat Systems-Ultrasonics/Misonix, Farmingdale, NY) set to 30% of maximum output power. Sonicated chromatin was then divided into aliquots for 3–4 immunoprecipitation (IP) reactions.

Each IP reaction was conducted using 1 µg of mouse monoclonal anti-AR (AR-441), mouse monoclonal anti-ERα (ERα D-12), or rabbit polyclonal anti-RORA (RORA H-65) antibodies (Santa Cruz Biotechnology, Santa Cruz, CA). Sonicated chromatin was also immunoprecipitated with normal mouse IgG or normal rabbit IgG antibodies (Santa Cruz Biotechnology) to serve as negative controls. Anti-RNA polymerase II (Millipore) was also included as a positive control. Immunoprecipitated DNA (IP-DNA) was then reverse-crosslinked to separate it from the proteins and purified using Millipore DNA purification columns (Millipore).

Real-Time qPCR analyses were conducted using an ABI Prism 7300 Sequence Detection System (Applied Biosystems) to measure the enrichment of each putative transcription factor binding site in the IP-DNA. Fold-enrichment of each binding site was determined by normalizing the Ct value of the transcription factor binding site in immunoprecipitated DNA with the Ct value of that transcription factor binding site in IgG-IP DNA. Primer sequences for qRT-PCR analyses designed by Primer 3 are listed in Table S2.

### Confocal immunofluorescence microscopy

Human tissue arrays from Dr. Janine LaSalle's laboratory (University of California, Davis) were obtained through the Autism Tissue Program (San Diego, CA). Each array contains 600 µm diameter×5 µm thick sections in triplicate from the BA9 region of the frontal cortex from autistic individuals and age- and sex-matched controls as well as from individuals with a variety of developmental disorders as previously described [26]. Confocal immunofluorescence analyses for RORA and aromatase proteins were performed using the Alexa Fluor-conjugated secondary antibody method of detection. Briefly, a tissue array was deparaffinized in xylene and rehydrated using a dilution series of 100%, 95% and 75% ethanol. The array slide was then subjected to heat-induced antigen retrieval by incubation in Tris-EDTA buffer (10 nM Tris Base, 1 mM EDTA, 0.05% Tween 20, pH 9.0) at 95°C for 25 minutes.

Rabbit polyclonal antibody (1∶50 final dilution) against RORA (4 µg/ml, Santa Cruz Biotechnology: sc-28612), goat polyclonal antibody (1∶50 dilution) against aromatase (4 µg/ml, Santa Cruz Biotechnology: sc-14245), and mouse monoclonal antibody (1∶100 dilution) against MAP2 (5 µg/ml, Invitrogen) were pooled and added to the tissue array in a humidified chamber. After overnight incubation at 4°C, unbound primary antibodies were removed by washing the slide 3 times with PBS. Alexa Fluor 488-conjugated donkey anti-mouse (1∶200 final dilution), Alexa Fluor 546-conjugated donkey anti-goat (1∶200), and Alexa Fluor 647-conjugated donkey anti-rabbit (1∶200) secondary antibodies (Invitrogen) were pooled and added to the slide for 45 min at room temperature. The slide was then washed and incubated with 4′, 6′-diamidino-2-phynylindole dihydrochloride (DAPI) for 5 min at room temperature to counterstain the nuclei. The slide was mounted using ProLong Gold antifade reagent (Invitrogen), and left in the dark at room temperature for 24 hrs before imaging. The fixed slide was imaged using a Zeiss LSM 710 confocal microscope (Carl Zeiss, Thornwood, NY) with a 60X objective lens. A total of 4 contiguous areas were scanned for each specimen, and the images were collected using ZEN 2009 software (Carl Zeiss). The same laser power and other scanning parameters were maintained for all specimens. Fluorescence signals from Alexa Fluor 546 (detecting aromatase) and from Alexa Fluor 647 (detecting RORA) are represented by red and blue pseudocolors, respectively. The MAP2 fluorescence, which was used to select neurons for RORA and aromatase quantification, was observed using green pseudocolor and DAPI nuclear stain was represented by gray (see Fig. S1).

Tissue images were coded with numbers and letters and analyzed in blind fashion using Volocity software (PerkinElmer, Waltham, MA). For each specimen, 40–50 frontal cortex neurons with distinct expression of MAP2 fluorescence and nuclear staining were selected for quantification of RORA and aromatase fluorescence intensities. The mean fluorescence intensity values from all neurons in each specimen were averaged and normalized by background subtraction.

### Statistical analyses

ANOVA analyses were performed to determine significance of the differences in expression levels between the hormonally-treated and untreated samples as well as the fluorescence intensities between the autistic and the control groups.

## Supporting Information

Table S1 **Potential transcription factor binding sites for AR, ER, and RORA.**
(DOC)

Table S2 **Primer sequences for RT-qPCR and qPCR analyses.**
(DOC)

Figure S1 **Confocal fluorescence images of brain tissues stained for A) nuclei, B) MAP2, C) Aromatase, and D) RORA.**
(TIF)
