# Supplementary material for: Sex Hormones in Autism: Androgens and Estrogens Differentially and Reciprocally Regulate RORA, a Novel Candidate Gene for Autism
Source: PLoS One. 2011 Feb 16;6(2):e17116. doi: 10.1371/journal.pone.0017116 (PMC3040206; doi:10.1371/journal.pone.0017116)
Supplement: Table S1 — Potential transcription factor binding sites for AR, ER, and RORA. (DOC) [file pone.0017116.s001.doc]

Table S2. Transcription factor binding sites on RORA and CYP19A1 genes
