# Supplementary material for: Sex Hormones in Autism: Androgens and Estrogens Differentially and Reciprocally Regulate RORA, a Novel Candidate Gene for Autism
Source: PLoS One. 2011 Feb 16;6(2):e17116. doi: 10.1371/journal.pone.0017116 (PMC3040206; doi:10.1371/journal.pone.0017116)
Supplement: Table S2 — Primer sequences for RT-qPCR and qPCR analyses. (DOC) [file pone.0017116.s002.doc]

Table S1. Primer sequences
