# Supplementary figures and images for: Sex Hormones in Autism: Androgens and Estrogens Differentially and Reciprocally Regulate RORA, a Novel Candidate Gene for Autism
Source: PLoS One. 2011 Feb 16;6(2):e17116. doi: 10.1371/journal.pone.0017116 (PMC3040206; doi:10.1371/journal.pone.0017116)

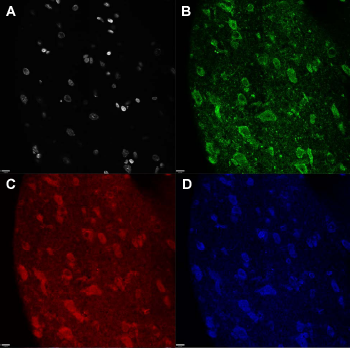

Supplement: Figure S1 — Confocal fluorescence images of brain tissues stained for A) nuclei, B) MAP2, C) Aromatase, and D) RORA. (TIF) [file pone.0017116.s003.tif]
